# Clowning in Health Care Settings: The Point of View of Adults

**DOI:** 10.5964/ejop.v12i3.1107

**Published:** 2016-08-19

**Authors:** Alberto Dionigi, Carla Canestrari

**Affiliations:** a Alberto Dionigi, Federazione Nazionale Clowndottori (FNC), Cesena, Italy; b Department of Education, Cultural Heritage and Tourism, University of Macerata, Macerata, Italy; Department of Psychology, University of Western Ontario, London, Canada

**Keywords:** clown, clown doctor, humor, positive emotions, complementary and alternative medicine, adults, well-being

## Abstract

Within the past decade, there has been a surge of interest in investigating the effects of clown intervention in a large variety of clinical settings. Many studies have focused on the effects of clown intervention on children. However, few studies have investigated clowning effects on adults. This paper presents an overview of the concept of medical clowning followed by a literature review conducted on the empirical studies drawn from three data bases (PubMed, PsycINFO, and Google Scholar), with the aim of mapping and discussing the evidence of clowning effects on non-children, namely adults. The following areas were investigated: Adult and elderly patients (mainly those with dementia), observers of clowning, namely non-hospitalized adults who are at the hospital as relatives of patients or health-care staff, and finally clowns themselves. The main results are that 1) clown intervention induces positive emotions, thereby enhancing the patient’s well-being, reduces psychological symptoms and emotional reactivity, and prompts a decrease in negative emotions, such as anxiety and stress; 2) clown doctors are also well-perceived by relatives and healthcare staff and their presence appears to be useful in creating a lighter atmosphere in the health setting; 3) few pilot studies have been conducted on clown doctors and this lacuna represents a subject for future research.

The history of clowning is a long and rich one. Not only do clowns provide the gift of laughter, but it is widely believed that they also have the power to bestow the gift of healing (Warren & Spitzer, 2013). For example, clowns are thought to have worked in hospitals since the time of Hippocrates, as the doctors of that time believed that humor had positive effects on one’s health (Koller & Gryski, 2008). In more recent times, the Fratellini Brothers, a famous clown trio, began working in French hospitals at the beginning of the 19th century: They occasionally visited hospitalized children to improve their moods (Warren & Spitzer, 2013). Today clowns have a greater presence in medical settings and play an important role within the health-care system. In recent years, interest has grown in the area of research on clown intervention in health-care settings: A significant number of empirical studies has been conducted under different clinical conditions and using different patient groups. To date, only two overviews of these studies have been published in English (Finlay, Baverstock, & Lenton, 2014; Sato, Ramos, Silva, Gameiro, & Scatena, 2016), and one has been published in Italian (Gremigni, 2014). These studies have specifically focused on the effect of clown interventions on children as primary beneficiaries; therefore, we decided to conduct an extensive and updated literature review of empirical scientific papers on clowns related to the work with adults in health-care settings: Moreover, aside from observations of the clown doctors themselves, our review includes discussions of the effects of clowning interventions on both patients and observers (e.g., health-care staff and relatives).

The presence of professional clowns as members of hospital health-care teams started in 1986, when Michael Christensen, a professional clown with the Big Apple Circus in New York, founded Big Apple Circus Clown Care (Koller & Gryski, 2008). The program’s aim was to prepare professional clowns to visit hospitals and to assist in the healing process through humor and clowning skills. These new characters, called *clown doctors*, parodied the work of medical doctors so that they would appear less scary to young patients. The clown doctors used circus skills, tricks, and improvisation while making their “rounds” to bring smiles and laughter to patients. Many clown care units (CCUs) were soon established in the United States, and analogous units began to spring up simultaneously in France, Germany, Britain, Italy, Spain, Switzerland, Austria, Canada, Australia, Brazil, and Israel (Ford, Courtney-Pratt, Tesch, & Johnson, 2014). State-of-the-art clowning in health-care settings assembles a diversity of practitioners, from well-intentioned volunteers to professional clowns, who have adapted their skills to serve patients undergoing medical care (Koller & Gryski, 2008). These practitioners, as well as child life specialists, are required to undergo comprehensive training through which they learn both artistic skills and strategies for dealing with psychological issues in the health-care system (Dionigi, Flangini, & Gremigni, 2012; Percelay et al., 2014).

Clowning in health-care settings represents a well-established way of entertaining children, adults, and the elderly during recovery (Dionigi, Ruch, & Platt, 2014). Clowning in health-care settings calls for a special way of interacting with patients and observers due to the variety of medical and emotional aspects involved; it therefore requires empathy and respect for each patient’s illness and psychological condition. When dealing with patients, clown doctors must be able to integrate artistic skills (e.g., music, comedy, mime, magic, or puppetry), which are useful in eliciting positive emotions, with personal qualities, such as empathy, emotional intelligence, and intuition. In this way, clown doctors can establish therapeutic relationships with patients and help to decrease their pain and other negative effects associated with their illnesses, as well as contribute to their well-being and create a lighter atmosphere (Finlay, Baverstock, & Lenton, 2014). Clown doctors generally conduct “clown rounds,” their version of medical rounds, which may prompt patients to forget their illnesses for a while. Nevertheless, clown doctors must be able to actively work in conjunction with hospital staff to design programs that meet the hospital’s needs. Several programs have been established over the years, such as those in which clowns visit both inpatients and outpatients, including those in intensive care, emergency rooms, and hematology/oncology units. In some programs, health-care clowns even accompany patients to the operating room (Warren, 2004).

Different work models for clowns in health-care settings have been established. For example, Big Apple’s model states that clown doctors must always work in pairs in order to support each other and to free patients from the pressure to participate (Linge, 2008). Another approach suggests that clowns should work alone, which may promote greater intimacy with patients (Koller & Gryski, 2008). Regardless of the approach used, how do clown interventions affect adults within the hospital setting? And with which adults do clown doctors interact? This paper aims to answer these questions by reviewing the literature on the topic.

## Literature Review

We first conducted a literature search in PubMed (using the publication dates January 1960–December 2015), since it is one of the most extensive and widely acknowledged databases used by science professionals with the aim to detect empirical studies that involved non-children. We used the following terms: “clown” OR “clown doctor” OR “clown therapy” OR “medical clowning” OR “hospital clown” OR “clinic clown.” The search terms were used for all fields (including title, abstract, keywords, and full text), and only papers written in English were included.

The search revealed 115 reports. We excluded 105 papers: Some were not relevant (70); some comprised theoretical issues (15); and others concerned empirical studies conducted primarily on children (20). Therefore, we reviewed a total of 10 empirical studies. The literature search proceeded with a focused exploration using the same terms as noted earlier, with the following preliminary criteria: 1) peer-reviewed full paper published in an international venue; 2) empirical study included; 3) studies published in English.

**Figure 1:**
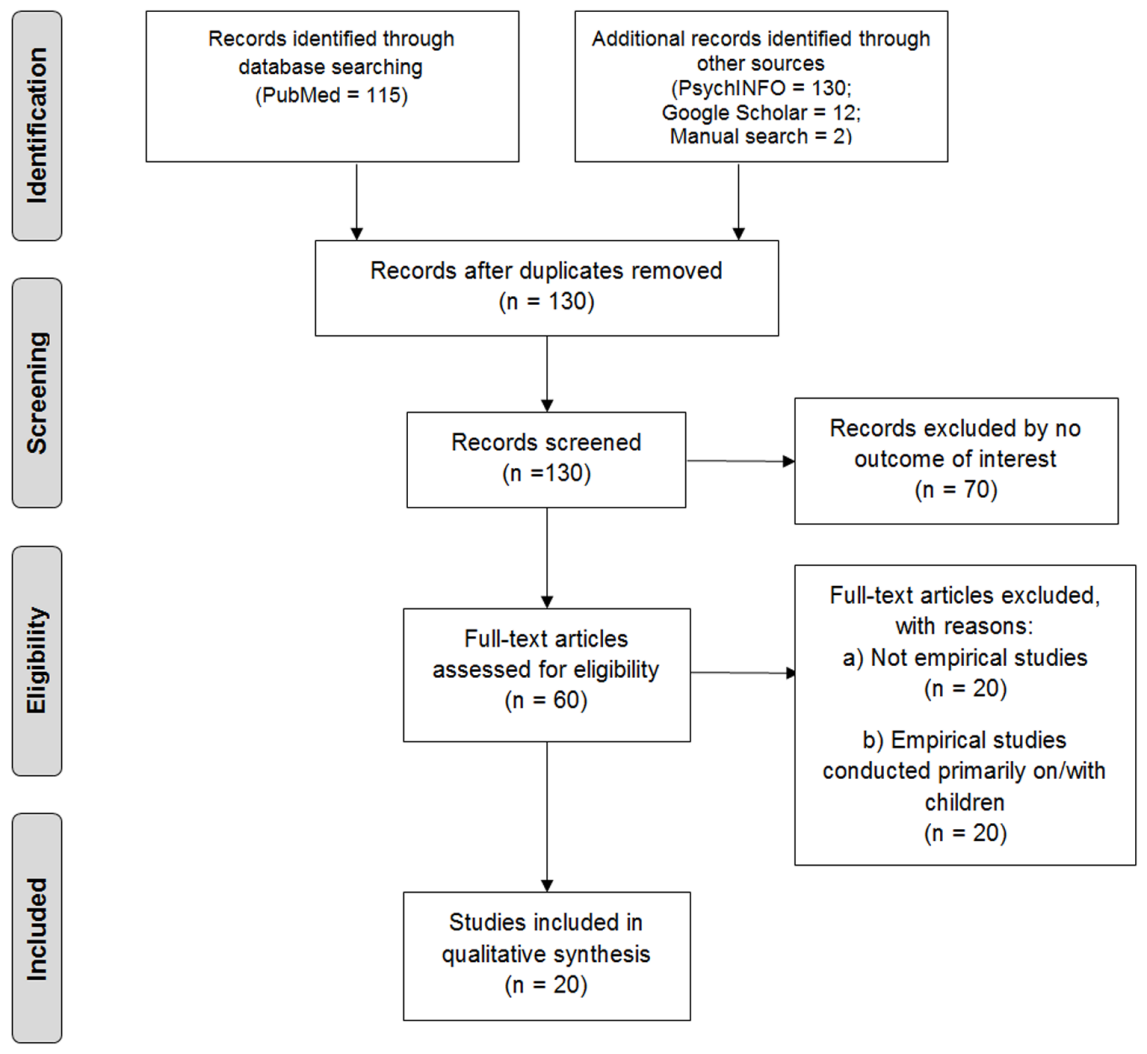
The flowchart summarizing the process of identifying the eligible studies.

The next step consisted of screening other databases in the following order (the number of new papers found in each library is indicated in brackets): PsycINFO (1) and Google Scholar (12). Finally, a manual search revealed two additional articles. During this step, 15 papers were found. The 25 papers selected were screened on the basis of an additional four inclusion criteria. To be included in the review, papers had to (a) include empirical evidence relating to the impacts and outcomes of clown intervention; (b) have been published during the period January 2005 to December 2015; c) include an abstract; and d) include participants over the age of 18 years. Using these four conditions, we then screened titles, abstracts, and (where possible) the full text of the manuscripts in order to exclude non relevant reports. Five reports were excluded, resulting in a final total of 20 papers that met the inclusion criteria and were identified as relevant to the current review. Details of the chosen papers are given in Figure 1.

## Results

Our literature review revealed that the 20 selected papers concerned one of the four following topics: 1) the effects of clown intervention on adult patients (for the most part, these studies mainly used experiments designed by the researchers); 2) research conducted on the elderly (mainly those with dementia); 3) the effects of clown intervention on health-care staff and relatives (these studies used either evaluative interviews or questionnaires); and 4) research conducted in respect of the psychological and artistic aspects of clowns. A brief summary of the main features of the studies reported in the 20 papers discussed is provided in Table 1.

**Table 1:** Details of the Reviewed Studies Chronologically Listed.

Author(s)	Sample	Aims/Objectives of study	Methods	Results and Conclusions
Angotti et al. (2015)	Nurses	To evaluate the role of clowns in decreasing nurses’ anxiety.	8 nurses (2 male and 6 female). Age Range = 30 – 49 (M = 40). Participants completed the STAI (Y1 and Y2) during the work shift, for a total of eight times each (4 times with clowns and 4 without).	Nurses’ anxiety was not reduced in the clown group and some nurses even showed increased anxiety when working with clowns.
Kontos et al. (2015)	Elderly patients with dementia	To examine elderly-clown practice and techniques based on qualitative interviews and ethnographic observations.	23 elderly participants with dementia (16 female, M = 87.8 years SD = 8.0) were involved in the study. A clown duo visited the residents twice a week over a 12-week period. Each clown duo–resident visit was video recorded to facilitate subsequent analysis.	The main important practice utilized by clowns was the “relational presence” that can be achieved through specific strategies between the clowns and the resident: (a) affective relationality; (b) reciprocal playfulness; and (c) co-constructed imagination.
Agostini et al. (2014)	Parents	To evaluate the efficacy of clown intervention in reducing preoperative anxiety in mothers whose children underwent surgeries.	25 mothers were included in the clown group (Age= 36.45 ± 5.71) and 25 in the non-clown one (Age = 35.95 ± 3.99). Mothers’ state and trait anxieties were measured at baseline and after separation from their children.	In the clown group the perceived stress and anxiety were reduced as well as anxiety and somatization did not increase after separation. compared with the non-clown group.
Auerbach et al. (2014)	Adults (external observers)	To identify emotional states induced in observers of hospital clown interventions by using a list of clown-specific ratings (CLEM-29).	**Study 1:** 119 adults (48.7% female) aged between 18 and 73 years (M = 30.66, SD = 13.53) watched videos of hospital clowns and circus clowns and completed a questionnaire to assess the elicited emotions. **Study 2**: 183 adults (26.8% male) with ages ranging from 18 -63 (M = 28.50; SD = 9.31) watched 15 videos of hospital clown interventions, circus clown performances, and nurse-patient interactions, and filled in a questionnaire to assess the elicited emotions.	**Study 1:** Results showed that general mood scales do not include emotional states elicited by hospital clowns. **Study 2**: amusement, transcendence, arousal, and uneasiness are the four factors which emerged. Circus clown and hospital clown performances elicited amusement. In addition, hospital clowns elicited feelings of transcendence, whereas nurse-patient interactions stimulated transcendent experiences but not amusement.
Dionigi et al. (2014)	Clowns	To develop a questionnaire able to capture the shift in and out from the clown character.	130 clowns (33 males and 97 females; age: 17 to 69 years) completed an online survey that included the Clown Shift Questionnaire and a demographic questionnaire.	Four dimensions influence the clown shift: positive beliefs, cognitive interference, reflective awareness, and anxiety. Positive beliefs and reflective awareness denote facilitating factors of the clown shift, while anxiety and cognitive interference hinder the process.
Ford et al. (2014)	Children, parents, Staff, clowns	To assess the impact of clown doctors’ activities on children, their families, staff and clown doctors.	Semi-structured, audiotaped interviews were conducted with 14 children (6 boys and 8 girls aged between 5 and 14 years) and their families. A Focus Group was conducted with 11 members of the staff.	Clowns provide an overall positive impact on the child in the moment of interaction (‘the encounter’) as well as during its anticipation.
Nogueira-Martins et al. (2014)	Nurse Students	To assess students’ perceptions about clown training.	70 students from different undergraduate courses of a healthcare university attended two 64-hour weekly hospital clown trainings.	Students were touched by the principles underlining hospital clowning that promote creative, respectful, and spontaneous relationships with others.
Tan et al. (2014)	Parents	To describe the benefits and barriers of clown care through a qualitative approach.	12 parents whose children received clown interventions in various hospital wards.	Clowns are perceived as bringing joy, happiness, laughter, amusement and a sense of meaningfulness in life. They can provide relief breaking long periods of hospitalization.
Leef & Hallas (2013)	Nurse Students	To examine the long-term effectiveness of the Sensitivity Training Clown Workshop (STCW).	131 nursing students took part in the workshop and 40 participants responded to an 18-month follow-up evaluation survey.	The workshop resulted useful to the majority of participants (employed in different areas), who reported that they usually apply lessons learned in the workshop in their practice.
Low et al. (2013)	Elderly patients	To evaluate the clown effect on a large group of elderly home residents.	Clown Group: 189 elderly patients in 17 nursing homes; Control Group: 209 residents in 18 nursing homes.	The humorous approach (both the humor session and the clown intervention) did not significantly reduce depression but significantly reduced agitation.
Barkmann et al. (2013)	Clowns, parents, staff	To provide an updated overview about hospital clowning in Germany and how clown intervention is perceived.	87 hospital clowns, 37 parents and 43 hospital staff members completed an online questionnaire regarding general conditions, procedures, assessments of effects and attitudes, as well as the Work Satisfaction Scale.	Clowns are well-trained, motivated and satisfied. Clown intervention can reduce stress in patients, and be useful both for parents and staff.
Blain et al. (2012)	Nurses	To assess clown perception by nurses working in a pediatric unit.	Semi-structured interviews were conducted on 13 nurses. The levels of physiological arousal, emotion and anxiety were measured in 9 out of 13 nurses under two conditions (the presence or absence of clowns.)	Eight nurses exhibited consistent changes in their response patterns when the clowns were present. Nurses' negative moods were reduced but no changes in anxiety were found. Qualitative data suggests that clown interventions also have a relational impact on nurses.
Hendriks (2012)	Patients with dementia	To illustrate the effect of clowns on patients with dementia from the point of view of the clowns themselves.	Clowns were required to report their activities and feelings experienced during their work.	The clown was able to bring pleasure and peace. The clown and the person with dementia were involved in a positive process of mutual articulation.
Nuttman-Shwartz et al. (2010)	Clowns	To evaluate the role of medical clowns with adult outpatients suffering from chronic illnesses.	Content analysis of the documentation of the work of two medical clowns over two years.	Three main dimensions were identified as important for the clown’s work: uncertainty about the definition of his role, lack of auxiliary skills, and appreciation of his intervention.
Friedler et al. (2011)	Adult pregnant patients	To evaluate the impact of medical clowning on pregnancy rates after in vitro fertilization (IVF) and embryo transfer (ET).	219 patients (110 in the intervention group and 109 in the control group) who underwent IVF and ET. Only women in the experimental group received clown intervention.	Pregnancy rate in the women who had clown intervention was significantly higher compared with the control group.
Brutsche et al. (2008)	Adult patients	To investigate the role of laughter in patients with severe chronic air flow obstruction (COPD) in reducing static lung volumes.	Patients with severe COPD (*n* = 19) and healthy controls (*n* = 10) received a clown intervention triggering regular laughter.	The intervention led to a reduction of total lung capacity (TLC) in the COPD group but not in the control group.
Koller and Gryski (2008)	Staff	To investigate the clown perception by parents and healthcare staff.	143 staff members and 51 parents filled in a questionnaire.	85% of staff appreciated the clown visits, and nearly 50% of them reported that the clowns supported their own work. Similar results were found in parents.
Linge (2008)	Clowns	To evaluate significant aspects about clown activity.	Descriptive analysis of reports after work by13 clowns (10 women and 3 men).	A relational pattern, characterized by empathic preparedness as well as a communication pattern, characterized by balanced synchronization of body language and verbal expressions were important.
Battrick et al. (2007)	Children, parents, staff	To elicit the perceptions of doctors, nurses, parents and children regarding the efficacy of performances by Clowns.	49 children, 43 parents/cares, 17 doctors and 93 other health-care staff filled in a questionnaire that included a mixture of closed and attitudinal Likert-type questions.	Medical doctors reported the positive role of clown doctors on sick children and their families’ mood. 83 out of 93 nurses agreed or strongly agreed that clown doctors have a positive impact on the child.
Higueras et al. (2006)	Adult psychiatric patients	To investigate the effects of a humor-based activity on behaviors in psychiatrics.	Patients in a psychiatric ward of a general hospital received clown interventions two days per week during two 83-day-long periods.	The intervention of the clowns reduced disruptive behaviors in general: attempted escape, self-injury, and fighting were significantly reduced.

### Clown Intervention on Adult Patients

Clinic clowning, which may on the surface appear to be only a children’s issue, is increasingly being applied in the care of adults and the elderly. One of the first studies on the efficacy of clowning in the treatment of adult patients was conducted in Switzerland: Brutsche, Grossman, Muller, Pello, Baty, and Ruch (2008) evaluated the effects of laughter induced by a clown in a group of patients with Chronic Obstructive Pulmonary Disease (COPD). A small group of patients (*n* = 19) with severe COPD and a control group (*n* = 10) were asked to complete the State-Trait Cheerfulness Inventory (STCI) and were tested using two pulmonary function tests, namely spirometry and plethysmography: The first one measures lung function as the volume and/or flow of air that can be inhaled and exhaled, while the latter measures the functional residual capacity of the lungs. A clown then performed for a mean duration of 30 minutes. During the intervention, the participants were videotaped for analysis through the Facial Action Coding System (FACS). The intervention led to a reduction of Total Lung Capacity (TLC) in the COPD group but not in the control group. The reduction of TLC was associated with a similar decrease in the reserve volume, indicating that the intervention reduced air trapping and so improved their lung function. Although results indicate a positive role of induced laughter in people affected by COPD, limitations such as the small sample involved, with people grouped in different sizes, the lack of randomization and of crossover design must be taken into account. Findings of this study need to be validated with larger samples and focused on a more reliable appreciation of the effect size and duration.

A study by Friedler et al. (2011) indicated that women entertained by a clown doctor after in vitro fertilization had more successful fertility treatments and increased pregnancy rates. This quasi-randomized study was conducted in an Israeli hospital and included 219 patients (110 in the intervention group and 109 in the control group) who underwent in vitro fertilization (IVF) and embryo transfer (ET). Only women in the intervention group received a clown visit after the ET. Each encounter lasted 12–15 minutes and included a routine based on jokes, tricks, and magic. Results showed that the pregnancy rate in the intervention group was significantly higher compared with that of the controls, perhaps due to stress reduction produced by the use of humor and medical clowning: Stress reduction, in fact, might improve fertility, resulting in positive effects on the engraftment. What seemed to work best was that, during the encounter with the medical clown, the patient was actively involved in the relationship in which she responds spontaneously to the ongoing interaction leading to a better involvement.

While the majority of studies conducted so far have investigated the role of clowns in reducing negative emotions, Auerbach, Hofmann, Platt, and Ruch (2014) focused on the positive emotions induced by clowns. The researchers conducted two studies to identify the emotional states induced in external observers via hospital clown interventions: In their first study, 119 adults (48.7% female) aged 18-73 years (*M* = 30.66, *SD* = 13.53), watched videos of hospital clowns and circus clowns and completed a questionnaire to assess the elicited emotions composed by the State-Trait-Cheerfulness Inventory (STCI-S<30>; Ruch, Köhler, & van Thriel, 1997), The Mood Rating Inventory (BSKE [EWL]; Janke, Hüppe, & Erdmann, 2003) and the 29 Clown Emotion List (CLEM-29; Auerbach et al., 2012). The authors found that several clown-specific experiences and judgments are not sufficiently well described in the existing models of emotional states and that the CLEM-29 could serve to fill this gap. In the second study, 183 adults (26.8% male) aged 18-63 (*M* = 28.50; *SD* = 9.31) watched 15 videos of hospital clown interventions, circus clown performances, and nurse–patient interactions, and completed questionnaires to assess the elicited emotions. The questionnaire responses showed that both circus and hospital clowns elicited amusement, but only the hospital clowns additionally elicited feelings of transcendence, constituted by feelings of being uplifted and surpassing the ordinary. Nurses elicited transcendence, because of the caring elements, but not amusement. Auerbach and colleagues tried to fill in a gap in the research in this field by providing an instrument able to evaluate the emotional states induced in observers of hospital clown. However, in this study, there was no real interaction with clowns: Non-patient adults watched clowning and nursing video clips. This lack of active relationship needs to be overcome because influencing mood-influencing factors (both positive and negative) are involved in face-to-face interactions.

### Clown Intervention on the Elderly and Patients With Dementia

Although few studies have been conducted on adult patients, research about clowns working in health-care has often focused on disabled patients, such as those affected by dementia or psychiatric diseases. Higueras et al. (2006) conducted a quasi-experimental study to examine the effects of clown doctors in decreasing the disruptive behaviors of psychiatric patients in a general Spanish hospital during two 83-day periods. During the first period (baseline), there were no interactions with clowns, while in the second period (intervention), patients were assembled in a common room and received two weekly 90-minute sessions of clown intervention. The efficacy of clown intervention was evaluated by the decrease of 10 behaviors considered to be disruptive, such as refusing to cooperate and shouting. Researchers then recorded the frequency of disruptive behavior (DB) during the day at three different time points. The results indicated that the intervention of the clowns reduced disruptive behaviors in general but that it reduced only three specific behaviors significantly (attempted escape, self-injury, and fighting). That was the first study conducted to evaluate the use of humor as a tool in therapy for severely ill patients. Results are not equivocal as only some DB decreased. Nevertheless some DB’s increased in frequency (such as refusal to cooperate and shouting) and authors related it to a higher disinhibition caused by the clown approach. However, the lack of control in the clown activity may have had an influence on this.

Clowning seems to also improve the quality of life of nursing home residents: Hendriks (2012) conducted an auto-ethnographic study in the Netherlands that focused on a special form of clowning for people who were at an advanced stage of dementia. The author revealed that the clown and the person with dementia were involved in a process of mutual articulation that helped patients to “be in touch” with their bodies. As a result of the clown activities, residents’ bodies became engaged in sensory conversations with other people. Although the results are intriguing, they lack systematic rationale as well as solid methodology as the analysis primarily concerns sensory conversations in the here and now. Future research should try to optimize and present robust data and results in order to generalize these.

Low and colleagues (2013) in a recent Australian study used a single-blind, two-group, longitudinal cluster randomized controlled study to evaluate the effects of clowning on elderly residents in 35 Sydney nursing homes. The intervention group comprised 189 elderly patients in 17 nursing homes; the control group consisted of 209 residents in 18 nursing homes. An integrated humorous approach that consisted of 1-day LaughterBoss training for each home’s nominated staff member, followed by 9 to 12 humor-therapy sessions by an Elder Clown, was used with the experimental group. Participants were assessed for depression using the Cornell Scale for Depression in Dementia (CSDD) and for agitation using the Cohen-Mansfield Agitation Inventory (CMAI) at three different times: Baseline (Week 0), post-session (Week 13), and follow-up (Week 26). The humorous approach (both the humor session and the clown intervention) did not significantly reduce depression but significantly reduced agitation. The authors pointed out that most of the subjects scored very low (floor effect) in relation to depression, as only 29% of the sample was assessed to have probable or possible depression, which could have influenced the results. The limitations of this study concerned variations between residents in the number of Elder Clown sessions they received and unbalanced baselines in several outcome measures between the two groups.

Kontos, Miller, Mitchell, and Stirling-Twist (2015) conducted a study to evaluate a 12-week Elder Clown program involving 23 residents of a dementia unit in a long-term care facility. The study involved 23 elderly participants (16 females and 7 males) who were primarily affected by Alzheimer’s disease (73.9%); the mean age was 87.8 years (*SD* = 8.0). Two clowns visited the residents over a 12-week period, and each visit lasted approximately 10 minutes. In order to facilitate the analysis, every clown visit was video-recorded. The main finding of the analysis was what the authors have defined as “relational presence.” This term captures “the reciprocal nature of engagement during plays, and the capacity of residents to initiate as well as respond to verbal, embodied, emotive, and creative engagement” (Kontos et al., 2015, p. 5). This peculiar presence is related to three core aspects: (a) affective relationality; (b) reciprocal playfulness; and (c) co-constructed imagination. Again, more than humor, in this study the opportunity to be “in touch” between the elder and the clown was the most important aspect. However, future research should focus on which aspects are primarily important in creating this bond. Finally, some criticism about this study arises regarding the use of the video camera that may have influenced individuals’ behavior in response to their awareness of being observed.

### Perceptions of Clown Intervention by Relatives and Health-Care Staff

The studies reviewed so far are related to clowning effects on patients, whether adults in general or elderly in particular, namely hospitalized people. Another set of empirical studies on -generally speaking- non-children, focuses on clowning effects on observers, namely non-hospitalized adults who are in hospital because they are relatives of patients or health-care staff. A Cochrane review (Yip, Middleton, Cyna, & Carlyle, 2011) showed that parental anxiety is common during a child’s hospitalization and surgery due to parents’ perception of the child’s worries and pain. Agostini et al. (2014) compared the anxiety rates of mothers whose children were scheduled to undergo general anesthesia for minor day surgery during the preoperative phase: 25 mothers and their children (*M* = 36.45; *SD* = 5.71) received the clown intervention (the experimental group), while the control group (25 mothers, *M* = 35.95; *SD* = 3.99) followed ordinary procedures, with no clown intervention. Results showed that there was a significant decrease in the scores of State-Trait Anxiety Inventory (STAI) related to the state of anxiety only in the clown-intervention group. The major limitations of this study are given by the small sample involved, having evaluated only the mothers, the lack of inclusion of objective physiological measures (e.g. heart rate and respiratory frequency) that could have provided more specific indicators about stress and anxiety.

Tan, Metsälä, and Hannula (2014) conducted a qualitative study at a Finnish university hospital for children in which 12 parents of children receiving clown care were asked to complete a semi-structured interview. Parents reported that the clowns helped to create a positive emotional state, as well as to promote interaction between them and their children. According to the participants, barriers to the clown intervention included timing and context, the psychological and emotional states of the children and parents, and the gravity of the illness. In this study there are two main limitations: It was carried out in Finland but interviews were conducted in English and this may have led some participants to refuse taking part in the interview both because they were not confident in using English, and because they had problems in articulating their thoughts. Again the small sample involved inhibits the possibility of generalizing the data.

Humor is considered to be a positive trait that may help nurses and doctors to cope with their work and to create a better atmosphere on the ward (Ruch, Rodden, & Proyer, 2011). Some studies on the interaction between clowns, patients, families, and staff have revealed a general acceptance of the clowns. Battrick, Glasper, Prudhoe, and Weaver (2007) conducted a study to test the perceptions of clowns by children, parents, doctors, and nurses in an English hospital. The majority of the 16 children’s doctors who participated in the study stated that clown doctors have a positive impact on sick children and their families, although six of the doctors reported that they did not personally like clowns. In addition, a questionnaire was sent to nurses: Of the 93 questionnaire returned, 83 nurses agreed or strongly agreed that clown doctors have a positive impact on the child and family. Again, a large number of nurses (*n* = 22) indicated that they personally did not like clowns.

In Australia, Ford et al. (2014) conducted a study aimed at investigating the impact of clowns on children, parents, staff, and on clown doctors themselves. The researchers utilized a mixed model consisting of observation, semi-structured interviews, and focus groups. This study revealed that clown doctor interactions had positive effects on all groups and the positive effects of clowning are not limited to the length of intervention. While children experienced anticipation and excitement before the clown visits, the clown’s props (e.g., balloons) may promote interactions, unrelated to hospitalization and illness, among children, family, and staff. Although this study also involved children we included it in the review as it provides useful information about the perception of clowns by relatives and staff. The study has a very simple rationale, and it would have benefited from an in-depth focus on the relationships among the various participants. Moreover, the positive outcome of the clown interventions is based on the skills of the performer and it is not easy to evaluate the positive effect systematically.

Koller and Gryski (2008) surveyed 143 staff members and 51 parents regarding the clown visits in a pediatric clinic in Toronto. Eighty-five per cent of the staff appreciated the clown visits, and nearly half of participants reported that the clowns supported their own work. Similar results were found for parents. In a German study (Barkmann, Siem, Wessolowski, & Schulte-Markwort, 2013), 87 hospital clowns, 37 parents, and 43 hospital staff members were recruited through an online survey aimed at clarifying the structural and procedural conditions of pediatric clowning. Both parents and staff indicated that they, as well as the patients, benefited from clown intervention. Results from this study are positive, questionnaires were developed for the study’s objective and frequencies about different appreciation variables were calculated. Again, the perception (in the two countries) is good and this speaks in favor of a greater inclusion of clowns in health settings. However, it would be helpful to have a higher correlation between the appreciation and psychological aspects (e.g. personality traits) for both clowns and participants.

A Canadian study by Blain, Kingsnorth, Stephens, and McKeever (2012) that involved 13 nurses investigated the effects of therapeutic clowns on hospitalized children. In addition to questionnaires, in nine cases, measurements of physiological arousal, emotion, and anxiety were obtained, both during the presence and the absence of clowns. Results showed changes in the automatic nervous system signals of eight nurses during the clown interventions. Moreover, the nurses’ negative moods were also reduced, although no changes in anxiety levels emerged. The main concern about this study lies in the small sample utilized and the lack of investigations of further personal variables such as the measures of emotional expressions and social interaction.

Two studies conducted in Brazil (Nogueira-Martins, Lima-Costa, Nogueira-Martins, & Nogueira-Martins, 2014) and in New York City (Leef & Hallas, 2013) investigated the role of clown training for undergraduate nurses: Students took part in clown workshops aimed at teaching nontechnical skills, such as improving emotional intelligence, empathy and communication skills. Nogueira-Martins et al. evaluated the perceptions of two groups of students (Group A, 40 participants; Group B, 30 participants) over a period comprising 64-hour weekly hospital clown trainings. Results showed that the training process was taken seriously and resulted in improvements in the nurses’ relationships with family, friends, and patients, as well as in the enhancement of oral presentations. Similarly, Leef and Hallas (2013) evaluated the efficacy of a Sensitivity Training Clown Workshop (STCW) provided to 131 baccalaureate nursing students. They conducted an 18-month follow-up evaluation survey: Based on the 40 questionnaires returned, it was possible to establish that the majority of participants appreciated and went on to apply competences acquired during the workshop. The two studies vary in length and participation, but similar results emerged: The clown training exhibited a potential for professional attitude construction and the development of interpersonal competencies. Unfortunately, in the first study, there was a high turnover of students that made generalizing the data difficult, while in the other it would have been useful to have had major information about the different specific aspects of participants. Taking part in a workshop on clowning has a great potential, but often people work on their limits and failures (see Lecoq, 2011) and this may lead to a strong refusal in taking part in the activity.

Angotti et al. (2015) investigated the role of clowns in decreasing nurses’ anxiety during children’s preoperative periods. The level of anxiety of eight nurses (two males and six females) aged 30-49 years (*M* = 40) was assessed via the STAI (Y1 and Y2) during the work shift, after preparing at least two children a total of eight times each (four instances with clowns and four without). Results showed that, compared to the control group, the nurses’ anxiety was not reduced but actually increased. This study shows specific limitations such as the very small number of participants and results which go in the opposite direction compared with the expected ones. Future research with larger groups must be conducted in order to improve understanding of the role of clowns in decreasing staff anxiety. It must be hypothesized that, since clown intervention is primarily directed towards the patients, nurses (even if involved) may not perceive the intervention as a coping strategy to deal with anxiety.

### Studies Conducted on Clowns: Psychological and Artistic Aspects

Research has mainly focused on the overall effects of hospital clowns. Although the importance of extensive training for clowns before entering the medical setting is acknowledged as important (e.g. Dionigi et al., 2012), little is known about the psychological characteristics of clown doctors. Some pilot studies have been conducted on clown doctors; however, they have included only small groups whose members often belong to the same Clown Care Unit (sharing a common training and operative model). Linge (2008) conducted a qualitative study on 13 clowns (three males and 10 females) in order to evaluate significant aspects of their activity: In this research, the artistic and psychological potential of working in pairs clearly emerged.

Nuttman-Shwartz, Scheyer, and Tzioni (2010) evaluated the content analysis of documentation regarding the work of two Israeli medical clowns with adult patients suffering from chronic illnesses. Medical clowns each worked twice a week for three hours; at the end of the day, each of them was required to document his/her activity and the emotions elicited by the encounters with the patients. Results showed that three elements emerged as important in determining the operative model: a) uncertainty about the definition of the clown role, as patients had difficulties in understanding this figure and in considering whether or not it was a part of the health-care staff; b) a lack of auxiliary skills, which led to the perceived frustration of clowns who had to cope with severely ill patients; and c) appreciation of the intervention, related both to the use of humor and to the ability to build relationships with patients. The two pilot studies show specific limitations due to the small number of participants, the relative restricted field of knowledge to be acquired and a lack of inter-difference in the approach utilized. However, the results may have an impact in developing more detailed and focused future research.

For example, starting with the assumption that a clown doctor capable of differentiating himself/herself from his/her playing a clown is more often perceived to be a good clown (Nuttman-Shwartz et al., 2010), Dionigi et al. (2014) designed a study aimed at capturing the essence of what it means to be a clown doctor. The authors claimed that assuming the clown role requires a cognitive shift that leads the clown doctor to perceive the world and to act in a different/unusual and foolish way, so that (almost) everything is permitted. The authors call this transition a “clown shift,” which represents the cognitive change that occurs when a person leaves the habitual state of mind to enter the clown’s state of mind and vice versa. People may differ in shifting in and out: Dionigi et al. (2014) studied which factors may promote or hinder the shift. A large sample of Italian clown doctors belonging to different associations (33 males and 97 females aged 17 to 69 years) participated in the study and completed an online survey that included the Clown Shift Questionnaire and a demographic questionnaire. Statistical analyses identified four dimensions as being significant in influencing this shift: Positive beliefs, cognitive interference, reflective awareness, and anxiety. Two of these (positive beliefs and reflective awareness) denote facilitating factors of the clown shift, while the anxiety experienced during the preparation and the presence of cognitive interference during the performance are two dimensions that hinder the process. Although this was the first study conducted on a large sample of clown doctors, some limitations need to be acknowledged: Firstly, even if the purpose was to define a questionnaire able to “capture” the real essence of the clown status quo, that was not completely achieved. Secondly, the study refers to data coming only from a specific population (Italian clown doctors) and future studies with different participants are needed.

## Discussion

Humor is an important part of life. In the last 30 years there has been a growing interest in its application in several contexts such as hospitals and homes, healthcare clowning now represents a well-defined approach. The small number of papers identified using our search terms confirmed that although there has been a surge of interest in healthcare clowning, as clowns are involved worldwide, little research has been conducted so far: Empirical evidence of such interventions is very sparse. A large amount of studies (not related to this paper’s aim) is related to studying the efficacy of clown interventions on children (see Finlay et al., 2014; Sato et al., 2016). One possible explanation is that clown intervention is generally addressed to children: Kids tend to be enthusiastic when encountering a clown who is able to capture their attention through comedy, gags, magic and props. Nevertheless, the first clown care unit (set up in New York) worked specifically with children. This may have led to imprinting the use of clowns in health settings. However, the healthcare clowning movement is increasing as are the application fields: Adults, the elderly, psychiatrics, healthcare staff are only some of the recipients of this approach, who have also received more attention from research.

Across the breadth of the literature reviewed, some studies have been conducted with the elderly or with patients suffering from psychiatric diseases: The core aspects of these studies were not restricted to the use of humor but also involved relational competences that every Healthcare Clown must possess. Clown intervention with adults and the elderly is a promising practice for improving the quality of life of recipients. From a broader perspective, in fact, the work of clown doctors aims to empower interpersonal relationships and to modify the atmosphere within the care setting by breaking up the seriousness of the setting with something positive, unexpected, and unconventional. Research should pay more attention to this field, as in recent years a large number of organizations is providing clown service to adult patients, especially elderly, psychiatric and disabled. So far results are positive, both when referring to the decrease of negative behaviors or negative emotions, and when evaluating the positive emotions elicited in recipients. The main gap in this respect is that only a few studies have focused on adults and on elderly and disabled people and the methodology used in these studies is very extensive and this makes comparing findings difficult.

A number of studies conducted on relatives of hospitalized patients has focused on assessing whether this approach may be useful to them as well as to children. The literature review presented here shows how clown intervention is helpful in reducing stress and anxiety in parents of hospitalized children, although this evidence is very sparse. However a very small amount of research is primarily dedicated to investigating parental emotional states (e.g. Agostini et al., 2014), while the majority of studies has been conducted in order to evaluate both the parental and healthcare staff perception of clowns, along with the children’s one. Results show that the clowns are well perceived and integrated into health settings, by different people. The practice of involving clowns in health settings is constantly increasing, thanks to the positive appreciation provided by patients, relatives, doctors, and nurses). This aspect challenges what was postulated by Vagnoli and colleagues (2005) who found that, although well perceived by doctors, clowns interfered with medical procedures. In this regard, a giant step forward has been taken with the increasing number of workshops aimed at introducing the role and functions of clowning within the healthcare setting to nurses, as well as involving them in the training in order to improve their relational skills.

Rigorous evaluations of the therapeutic effects of clowning are complex, as clowning is a multimodal intervention that is set according to medical conditions, procedures, family functioning, and health-care teams (Ford et al., 2014). Moreover, clown intervention covers a large variety of activities: During their performances, clown doctors are required to adapt their techniques while keeping in mind that their goal is to change the emotional state of the patient and to improve the patient’s environment.

Finally, there is a paucity of studies about clown doctors themselves: Two out of the three studies evaluated in this literature review are qualitative studies and have been conducted on very small samples (two and 13 clowns). However, these two studies provided the basis for a subsequent research about important aspects for playing the role/dressing the part of the clown (Dionigi et al., 2014) even though specific limitations emerged about the construction of the main questionnaire and the sample involved. In this regard, further research should be undertaken to shed light on the psychological and artistic skills of clown doctors, which may contribute to identify those characteristics required to hire the best candidate with the potential to lead to positive patient interventions.

To sum up, research in the field of healthcare clowning is very young: The first published article evaluating the efficacy of a clown intervention in decreasing children’s preoperative anxiety dates back to 2005 (Vagnoli et al., 2005). Since then there has been a growing number of studies, and these studies concerning the efficacy of clown doctors have indicated a general effectiveness in several fields. However, according to the presented results, further research should focus more on adult patients, within different hospital departments (such as oncology, orthopedics, etc.) on elderly and disabled people, where clowns have proven to be effective in inducing positive emotions, as well as on the perception of relatives and healthcare staff. In this regard, it would be important to set protocols and standardized approaches founded on evidence-based results in order to establish well-defined guidelines for different patients.

From a broader perspective, the work of clown doctors aims to empower interpersonal relationships and to modify the atmosphere within the care setting by bringing something positive, unexpected, and unconventional (Warren & Spitzer, 2013). Although research is progressing, there is still a gap in determining which aspects are more relevant in leading to positive results. Undoubtedly, humor is an important aspect but not the only one: Further studies should consider which features of affectivity are induced, as well as which positive emotions are elicited during the different gags.
